# Trojan Horse‐Like Nano‐AIE Aggregates Based on Homologous Targeting Strategy and Their Photodynamic Therapy in Anticancer Application

**DOI:** 10.1002/advs.202102561

**Published:** 2021-10-20

**Authors:** Yin Li, Rongyuan Zhang, Qing Wan, Rong Hu, Yao Ma, Zhiming Wang, Jianquan Hou, Weijie Zhang, Ben Zhong Tang

**Affiliations:** ^1^ AIE Institute State Key Laboratory of Luminescent Materials and Devices Center for Aggregation‐Induced Emission Key Laboratory of Luminescence from Molecular Aggregates of Guangdong Province South China University of Technology Guangzhou 510640 China; ^2^ Department of Urology The First Affiliated Hospital of Soochow University 188 Shizi RD Suzhou 215006 China; ^3^ School of Materials Science and Engineering Nanchang Hangkong University Nanchang 330063 China; ^4^ Department of Urology Dushu Lake Hospital Affiliated to Soochow University Suzhou 215006 China; ^5^ Shenzhen Institute of Aggregate Science and Technology School of Science and Engineering The Chinese University of Hong Kong Shenzhen Guangdong 518172 China

**Keywords:** aggregation‐induced ROS generation, homologous targeting, photodynamic therapy, therapeutic mechanism

## Abstract

Photodynamic therapy (PDT) has become a promising candidate for cancer theranostics; however, traditional photosensitizers (PSs) usually exhibit weak fluorescence and poor reactive oxygen species (ROS) generation efficiency when aggregated. Recently, aggregation‐induced emission (AIE) luminogens have shown great potential in the development of novel PSs owing to their excellent aggregation‐induced ROS generation (AIG‐ROS) activity. However, there are still concerns that must be addressed. In this study, two near‐infrared (NIR) emitters (PI and PTI) are synthesized with AIG‐ROS characteristic. PTI exhibit a valuable redder emission with more effective intersystem crossing (ISC) process than PI. The two AIE‐active PSs show excellent lipid droplet (LD)‐specific targeting ability. The detailed therapeutic mechanism of PDT in LDs specificity is also investigated. The mechanism of oxidation of polyunsaturated fatty acids (PUFAs) in LDs to form toxic lipid peroxides (LPOs) and thereby causing cellular ferroptosis is confirmed first. Homologous targeting is also used to achieve tumor targeting via coating PSs with active cancer cell membranes. Biomimetic aggregates exhibit good targeting ability, and an improved PDT antitumor effect via AIG‐ROS activity. These findings offer a clear route to develop advanced PSs with good targeting specificity. A template has also been provided for studying the therapeutic mechanism of AIE‐active PSs.

## Graphical abstract

The process of photodynamic therapy in the lipid droplets becomes clear via cellular ferroptosis caused by singlet oxygen species oxidating polyunsaturated fatty acids to form toxic lipid peroxides, and the strategy of homologous anticancer targeting is employed to achieve precise tumor targeting, which exhibits better suppression and even elimination of tumor growth.

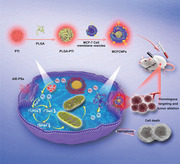

## 1 Introduction

Photodynamic therapy (PDT)^[^ ^1^, ^2^, ^3^, ^4^ ^]^ has become an effective and developing cancer treatment modality due to its advantages of noninvasiveness, high spatiotemporal accuracy and controllability, minimal drug resistance, and low biological toxicity and immunostimulatory activity. These benefits overcome the various side effects of traditional modalities for cancer therapeutic (such as surgical resection, radiotherapy,^[^ ^5^ ^]^ and chemotherapy^[^ ^6^, ^7^ ^]^). Consequently, PDT has attracted the attention of many researchers in recent years.^[^ ^8^, ^9^, ^10^ ^]^ Under light irradiation, photosensitizers (PSs) transfer electrons or energy to surrounding molecular oxygen through photochemical reactions to generate reactive oxygen species (ROS) with strong phototoxicity, which can kill tumor cells and activate antioncogenes.^[^ ^11^, ^12^, ^13^, ^14^ ^]^

Conventional PSs exhibit weak fluorescence when aggregated; however, aggregation‐induced emission luminogens (AIEgens) have recently become very popular because of their good luminescence properties and aggregation‐induced generation of ROS (AIG‐ROS) activity.^[^ ^15^ ^]^ The benefits of these AIE‐active PSs have been demonstrated, but there are some associated problems that must be solved. First, a redder emission of AIEgens is expected to be achieved with a more effective intersystem crossing (ISC) process from the excited singlet state to the triplet platform to boost ROS generation.^[^ ^16^, ^17^ ^]^ Second, the mechanism of the PDT process based on AIEgens needs to be clarified to enable finer molecular design and more effective discussion of the cellular information pathway.^[^ ^18^, ^19^ ^]^ Additionally, the key problem in practical application is how to target high‐efficiency luminous AIEgens to tumors.^[^ ^20^, ^21^, ^22^, ^23^, ^24^ ^]^

**Figure 1:**
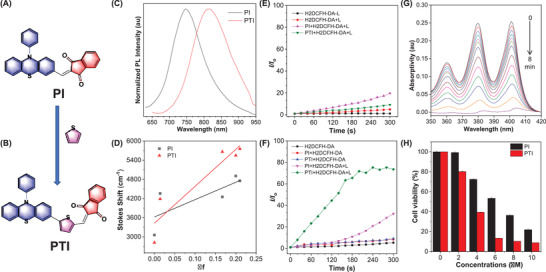
Chemicalstructures of A) PI and B) PTI. C) PL spectra of PI and PTI in FEAT FILM. D) Correlation of solvent polarity parameter with Stokes shift. The total ROS generation efficiency of PI and PTI. Plots of the relative PL intensity at 525 nm of H2DCFH‐DA in E) DMSO and F) PBS upon white light irradiation (50 mW cm^−2^) for different times. G) Absorbance intensity of ABDA after photodecomposition by ROS upon white light irradiation of PTI. H) Viability of MCF‐7 cells incubated with different concentrations of PI and PTI upon white light irradiation (50 mW cm^−2^). Concentrations: 10 × 10^−6^ m (AIEgens), 20 × 10^−6^ m (H2DCFH‐DA), and 40 × 10^−6^ m (ABDA).

To solve the above problems, in this study, PI was designed as an AIE‐active model PS with near‐infrared (NIR) emission and a good ROS generation efficiency via electronic donor and acceptor (D–A) molecular engineering. A thiophene ring was then introduced into the skeleton of PI to obtain PTI to enhance the ISC process by the heavy atom effect (**Figure** **1**A,B). With redder emission, more efficient ROS generation was also realized. In addition, the two PSs targeted lipid droplets (LDs) with similar AIG‐ROS features. The cell death mechanism underlying the PDT process in LDs was clarified by monitoring the levels of intracellular glutathione (GSH) and glutathione peroxidase 4 (GPx4), which were produced as a result of ferroptosis via oxidation of polyunsaturated fatty acids (PUFAs) to toxic lipid peroxides (LPOs). Additionally, homologous anticancer targeting was used to efficiently promote the phagocytic behavior of cells and achieve precise tumor targeting. Tumor cell membrane was used to coat PTI aggregates to prepare MCFCNPs, which suppressed tumor growth better and even eliminated tumors. Therefore, the study is the first to use biomimetic nanotechnology to increase AIG‐ROS for efficient and tumor‐specific PDT. It is also the first to reveal the potential of AIE‐active PSs as next generation imaging and theranostic agents.

## 2 Result and Discussion

### 2.1 Photophysical Property

The detailed synthetic routes of PI and PTI are illustrated in Figure S1 (Supporting Information). Nuclear magnetic resonance and high‐resolution mass spectrometry were used to confirm the molecular structures of PI and PTI. The two target products had good solubility in tetrahydrofuran, dichloromethane, ethyl acetate and other common organic solvents; however, they were not soluble in water and ethanol. Two absorption bands were observed for every emitter in the unimolecular state, and the larger band at ≈500 nm was attributed to the intramolecular charge transfer (ICT) transition. For the high‐energy absorption peaks, PTI (397 nm) exhibited an obvious redshift compared to PI (343 nm) due to the extended electronic conjugation skeleton created by thiophene ring (Figure S2, Supporting Information), which was consistent with the result of the theoretical calculation results (Figure S3, Supporting Information). Deep red and NIR emission with maximum peaks at 667 nm and 700 nm for PI and PTI respectively in solution were shown (Figure S2, Supporting Information). A similar red effect was observed for PTI. In the solvation experiment, the gradually redder emission displayed special sensitivity to increasing solvent polarity (Figures S4 and S5, Supporting Information). However, the photoluminescence quantum yields (PLQYs) decreased, suggesting a typical ICT excited state with special polarity sensitivity and a stronger ICT effect by PTI compared to PI (Figure S6 Supporting Information and Figure 1D). Interestingly, the photoluminescence spectra in the neat film showed a redshift the especially for PTI. Additionally, the maximum emission peak for PTI was located at 813 nm, and the tail of the emission peak exceeded 900 nm, demonstrating the NIR‐I area (Figure 1C), which had potential for application in NIR‐I fluorescent imaging. Similar AIE behaviors of PI and PTI were observed at different water fractions (*f*_w_), which were all higher than their PLQYs in dimethyl sulfoxide (1.7% and 0.6%) respectively (Table S1 and Figure S7, Supporting Information).

### 2.2 Cell Imaging

Cell imaging experiments were conducted as a preliminary biological study. MCF‐7 cells were first preincubated with oleic acid (OA) to induce a considerable amount of neutral lipids, and then PTI and PI at specified concentrations were incubated with these MCF‐7 cells. As shown in Figure S8 (Supporting Information), PI showed a better cellular permeability than PTI because most of PI entered the cells with strong red fluorescence. This difference in cellular levels of the fluorescent labels can be attributed to the twisted molecular structure of PI. To demonstrate the specific cellular location stained by these emitters, a commercial boron fluoride dipyrrole (BODIPY) probe for specific LD imaging was coincubated with PTI and PI (Figure S9, Supporting Information). The Pearson's correlation coefficient for PI was 0.94, which was higher than that for PTI (0.79), demonstrating that most of the AIEgens in the cells were located in LD‐targeted organelles because of their polarity.

### 2.3 Light‐Triggered ROS Generation and PDT In Vitro

Usually, a heavy atom effect and strong ICT effect are beneficial for ISC channels to generate excited triplet energy, providing the potential to boost toxic ROS generation to suppress tumor growth in PDT. Low‐temperature phosphorescent spectra were characterized to reflect the effective production of triplet energy after photoexcitation (Figure S10, Supporting Information). The energy splitting (Δ*E*_st_) between the lowest singlet and triplet states of PI was 0.13 eV, whereas that for PTI was 0.03 eV, which predicted a more efficient ISC process for the PTI emitter according to perturbation theory.^[^ ^25^ ^]^ Therefore, PTI was expected to induce a higher ROS production.

The H2DCFH‐DA probe was generally used to evaluate ROS production efficiency. After irradiation with safe white light, PTI and PI showed low ROS generation efficiency in dilute conditions (Figure 1E). However, in aggregates, the fluorescence intensity of H2DCFH‐DA increased rapidly to nearly 80 times that of PTI, which was much greater than that of PI (30 times). This suggested that PTI had a better ROS generation efficiency than PI (Figure 1F). The AIG‐ROS effect of AIE‐active PSs was confirmed again.

In addition, the absorbance of the ABDA probe decreased significantly mixed with PTI and PI under white light irradiation, demonstrating that singlet oxygen species were generated (Figure 1G and Figure S11, Supporting Information). Toxicity evaluation in vitro was carried out, and these two PSs showed excellent biosafety without light irradiation (Figure S12, Supporting Information). However, the cell viability obviously reduced when the incubated concentration increased under light irradiation, which indicated that the PDT was effective (Figure 1H). Notably, PTI showed a better anticancer effect than PI, which was consistent with the ROS generation efficiency.

### 2.4 Therapeutic Mechanism

It is reported that most AIE‐active PSs destroyed lysosomes or mitochondria via cellular apoptosis, necrosis, autophagy, or paraptosis by toxic ROS;^[^ ^18^, ^26^, ^27^ ^]^ however, there is little discussion about the therapeutic mechanisms underlying the antitumor effects of LD‐targeting AIE PSs. In general, cellular ferroptosis caused by the oxidation of PUFAs in LDs by singlet oxygen species to produce LPO was the main mechanism by which death occurs as a result of LD biotoxicity. ^[^ ^28^, ^29^ ^]^

Phosphatidylcholine (PC) and OA were used to simulate the environment of cellular LDs to demonstrate the ability of PTI and PI to oxidize LDs in PDT by mass spectrometry analysis (Figure S13, Supporting Information). The molecular weights (*M*_W_) of oxidized PCs were significantly higher than the *M*_w_ of the pure PC reference by 16 (one oxygen atom) and 64 (four oxygen atoms), associated with the two double bonds (CH═CH) in the PC alkyl chain being oxidized to form lipid oxide (LO) or lipid peroxide (LOO). The *M*_w_ of OA also increased by 16 (one oxygen atom) and 32 (two oxygen atoms) after oxidization, suggesting that the CH═CH in its alkyl chain was oxidized to form LO or LOO.^[^ ^30^, ^31^, ^32^ ^]^ The results showed that PUFAs in LDs could be oxidized to generate LPOs during PDT. Therefore, the specific probe Liperfluo was used to validate LPO generation.^[^ ^33^, ^34^ ^]^ The results showed that the fluorescence intensity from Liperfluo in the cells was clearly triggered by LPO generation during incubation with PTI and PI after light irradiation (Figure S14, Supporting Information).

**Figure 2:**
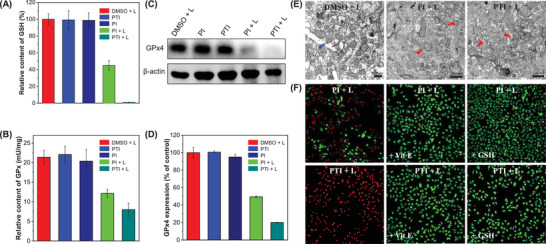
PDT of MCF‐7 cells with PI and PTI, relative content of A) GSH and B) GPx. C) Western blot detection of GPx4. D) Gray quantitative histogram of (C). E) TEM imaging after PDT treatment; red triangle arrow points to normal mitochondrial morphology, blue triangle arrow points to the damaged mitochondria morphology. F) Fluorescence imaging of Calcein‐AM‐ and propidium iodide‐stained MCF‐7 cells after different PDT applications. Concentration: 10 × 10^−6^ m (AIEgens), DMSO: 0.1–0.3%). Scale bar = 500 nm for TEM.

GSH depletion and GPx4 inactivation are two pivotal indicators of ferroptosis, which is influenced by the content of LPO in cells. In general, GPx4 catalyzes the conversion of toxic LPOs produced in cells into nontoxic lipid alcohols with GSH serving as a necessary cofactor.^[^ ^35^ ^]^ When ROS production increased in cell, these is an excessive accumulation of LPOs, which triggers ferroptosis via GPx4 inactivation and GSH depletion.^[^ ^36^ ^]^ GSH content in the MCF‐7 cells decreased by more than 50% when the cells were subjected to PDT with PI. However, GSH was completely depleted in the PTI group after PDT. This was attributed to PDT‐induced increase in ROS levels, which caused inactivation of the GPx enzyme system (**Figure** **2**A,B). In addition, the two PSs reduced the total expression of GPxs, especially the expression of GPx4 (Figure 2C,D). These results demonstrated that the balance between oxidative stress and antioxidant mechanisms was disrupted by GSH depletion. Additionally, the reduced GPx4 expression resulted in increased LPO accumulation, which built up the material needed for ferroptosis.

Ferroptosis is evidenced by changes in mitochondria, such as a reduction in mitochondrial size, with condensed membrane densities and a reduction or elimination of mitochondrial cristae.^[^ ^37^, ^38^ ^]^ As shown in the transmission electron microscopy (TEM) images (Figure 2E), abnormal mitochondria lacking cristae (red arrow) were observed after PDT, in contrast to normal mitochondrial morphology with distinct cristae (blue arrow), indicating ferroptosis in most of the cancer cells. Calcein‐AM and propidium iodide were used as fluorescent indicators of live/dead cells. The results showed that almost all the cells in the PTI group died after PDT, but the antioxidants (vitamin E or GSH) maintained better cell viability in spite of the PDT (Figure 2F). Hence, the mechanism of ferroptosis was confirmed for these two new AIE‐active PSs.

### 2.5 Targeted Imaging and PDT In Vivo

**Figure 3:**
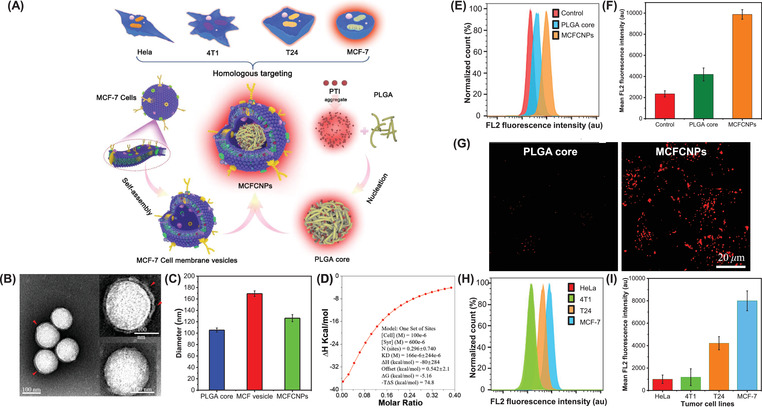
A) Preparation procedure of MCFCNPs. Extracting cancer MCF‐7 cell membrane and then coated onto PTI‐loaded PLGA cores by electrostatic adsorption interaction. Characterization of biomimetic MCFCNPs. B) TEM images of MCFCNPs; C) the particle size of PLGA cores, MCF‐7 cell membrane vesicles, and MCFCNPs; D) isothermal titration calorimetric curve of MCF‐7 cell membrane vesicles binding to PLGA cores; CLSM images and flow cytometric profiles of E) the uptake rate of MCF‐7 cells and F) mean fluorescence intensity (MFI) treat with different nanoparticles including PTI, PLGA cores, and MCFCNPs; G) CLSM images of MCF‐7 cells treat with PTI core and MCFCNPs (Concentrations: 10 × 10^−6^ m (NPs)); H) the uptake rate and I) MFI of four cell lines including Hela, 4T1, T24, and MCF‐7 cell coincubation with MCFCNPs for 2 h.

To further improve the efficiency of ROS production through AIG‐ROS property and simultaneously endow PSs with tumor targeting properties, a homologous targeting strategy with advantages of excellent biocompatibility and fast cytophagy was adopted by coating the PSs with MCF‐7 cell membrane. **Figure** **3**A showed how PTI was used to synthesize MCFCNPsr, and the method used was described in the supporting information. The TEM images showed that the solid core surface of the MCFCNPs was covered with a gray film‐like material to form a core‐shell structure (Figure 3B). The emission peak of the MCFCNPs had a blueshift relative to the PTI, which was caused by noncovalent bonding between the fluorogen and proteins through hydrophobic and van der Waals interactions (Figure S15, Supporting Information).^[^ ^39^, ^40^ ^]^ Dynamic light scattering (DLS) analysis revealed that the particle size and zeta potential of the MCFCNPs were ≈120 nm and −22 mV, respectively, which were within the ranges obtained for the poly(lactic‐*co*‐glycolic acid) (PLGA) cores and MCF‐7 cell membrane vesicles (Figure 3C and Figure S16, Supporting Information). Evaluation of changes in the size of the MCFCNPs after 96 h shows that the particles were very stable (Figure S17, Supporting Information). Furthermore, the amounts of epithelial cell adhesion molecule, N‐cadherin and galectin‐3 on the surface of the MCFCNPs were not significantly different from those on the surface of MCF‐7 cell membrane vesicle proteins, which proved that the nanoparticles (NPs) had been successfully prepared (Figures S18 and 19, Supporting Information). These proteins imparted a bionic function to the MCFCNPs, which was a prerequisite for homologous targeting and increased intratumoral enrichment. Isothermal titration calorimetry (ITC) characterization demonstrated that electrostatic adsorption was the major driving force that connected the cell membranes to the PLGA cores (Figure 3D and Figure S20, Supporting Information).

The homologous targeting ability of these MCFCNPs was tested. Flow cytometric analysis revealed that cells incubated with MCFCNPs had the highest uptake of NPs. Consequently, their fluorescence intensity was stronger than that of the control (PTI) group or PLGA core group (Figure 3E,F), demonstrating that MCFCNPs were more easily recognized and taken up by the cells than bare PLGA cores or PTI were. Confocal laser scanning microscopy images also revealed that uptake of the MCFCNPs via endocytosis was faster than that of the PLGA core, as the cells showed a higher red fluorescent brightness with the MCFCNPs (Figure 3G). Afterward, MCFCNPs were incubated with different cells (HeLa, 4T1, T24, and MCF‐7 cells) to investigate homologous targeting ability. As shown in Figure 3H,I, homologous MCF‐7 cells took up the most NPs and displayed the strongest fluorescence signal. This further confirmed that MCFCNPs had excellent specific binding ability with homologous MCF‐7 cells, thus indicating that MCFCNPs had homologous targeting and promoted its uptake by homologous tumor cells.

**Figure 4:**
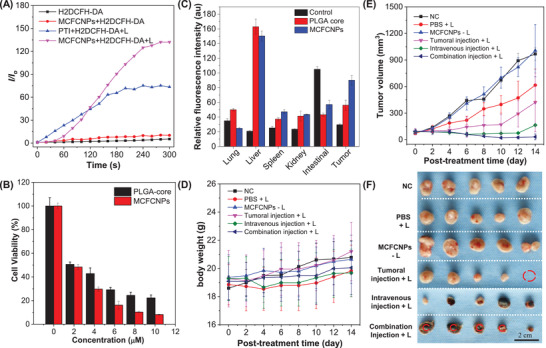
A) Plots of the relative PL intensity of H2DCFH‐DA in PBS upon white light irradiation (50 mW cm^−2^) for different times. B) Viability of MCF‐7 cells incubated with different concentrations of PLGA core and MCFCNPs for 12 h, then irradiated with white light (50 mW cm^−2^) for 10 min. Concentrations: 10 × 10^−6^ m (AIEgens) and 20 × 10^−6^ m (H2DCFH‐DA). C) Semiquantitative analysis of the relative fluorescence intensities of isolated tumor and organs (lung, liver, spleen, kidney, and intestinal) in different experimental groups (injection with PBS, PLGA core, and MCFCNPs). In vivo PDT efficacy of homologously targeting MCFCNPs to MCF‐7 tumor‐bearing nude mice. D) The body weight of mice after different treatments for 14 d. E) MCF‐7 tumor growth curves of different groups after treatments (*n* = 14). F) Photos of the tumors collected from the mice after PDT treatment. The red circle in the figure represents the cure of the tumor.

As a proof‐of‐concept, the ROS generation efficiency of MCFCNPs with better homologous targeting ability was characterized. As shown in **Figure** **4**A, compared to the bare PTI group in phosphate‐buffered saline (PBS), the NPs coated with MCF‐7 cells membrane induced a higher ROS production. This suggested that the homologous targeting strategy resulted in the PSs showing excellent targeting ability and improved ROS generation efficiency to strengthen the therapeutic effect of PDT. Notably, MCFCNPs entered the cells within 50 min, which significantly accelerated cellular uptake of the biomimetic PSs coated with the biocompatible cellular membrane, and a brighter red fluorescence could be observed with the incubation time extended to 190 min (Figures S21 and S22, Supporting Information). PTI coated with cellular membrane and PLGA had negligible cytotoxicity (Figure S23, Supporting Information); however, most of the cells that were incubated with a low‐concentration of MCFCNPs and PLGA NPs were killed after white light irradiation, indicating excellent PDT efficiency in vitro (Figure 4B). In a further study, the PDT efficiency of the MCFCNPs was compared to that of pure PTI. The results of the experiment showed that the half‐maximal inhibitory concentration of the MCFCNPs against the MCF‐7 cells viability was 1.9 × 10^−6^ m that of pure PTI was 3 × 10^−6^ m, which reflected improved therapeutic efficiency with the cell membrane coating. Therefore, tumor treatment in vivo with excellent efficiency was anticipated due to the improved biocompatibility and ROS production by the MCFCNPs.

Tumor imaging and treatment were carried out of MCFCNPs in vivo to evaluate the imaging and therapeutic efficacy of MCFCNPs in nude mice bearing MCF‐7 tumors. After intravenous injection of MCFCNPs via the tails of the mice, the fluorescence intensity at tumor location was found to be satisfactory (Figure 4C). Tumor volume and body weight were measured every 2 d after treatment within 14 d to evaluate the therapeutic effect of the various formulations. There was no obvious variation in mouse weight in all the treated groups, which suggested that the treatments were well tolerated (Figure 4D). The growth curves and the tumor images showed that the treatments administered to be the three untreated control groups (negative control, PBS and MCFCNPs without irradiation) had no apparent efficacy in suppressing tumor growth. In contrast, the tumor decreased significantly in size and even disappeared after 14 d following intratumoral injection of the MCFCNPs, which shown the strong therapeutic effect of the MCFCNPs. In addition, the combination of intratumoral injection and tail vein injection of the drugs resulted in a stronger therapeutic effect compared to single drug injections (Figure 4E,F and Figures S24 and S25, Supporting Information).

### 2.6 Pharmacokinetic Study and Biosafety Assessment of MCFCNPs In Vivo

The pharmacokinetic studies showed that the blood half‐life of the MCFCNPs was about 6 h, which was beneficial for MCFCNPs accumulation in the tumors by the enhanced permeability and retention effect for fluorescence imaging and photodynamic therapy in vivo. The fluorescence intensity returned to the preinjection level after 12 h, indicating that metabolism of the MCFCNPs was completed within 12 h (Figure S26, Supporting Information). Ex vivo fluorescence images of main organs and tumors excised from the mice at 24 h after intravenous injection of MCFCNPs or PLGA were obtained and evaluated. PBS was used as the control treatment in the study. In the MCFCNPs‐treated group, the fluorescence signal of tumors was strong; however, it was weaker than that of the liver, which could be attributed to the reticuloendothelial system,^[^ ^41^, ^42^ ^]^ but kidneys, heart, spleen, lungs and intestines showed only weak fluorescence signals (Figure S27, Supporting Information). Moreover, the fluorescence intensity of tumors in the MCFCNPs‐treated group was stronger than that in the PLGA group, indicating that the MCF‐7 cell membrane facilitated accumulation of the PSs in the tumors.

The potential toxicity or side effects of the PSs were further explored in healthy nude mice after MCFCNPs were intravenously injected into the mice at a high dose. PBS was used as the control treatment in this experiment as well. After 14 d, the mice were sacrificed and their blood and other tissues were collected for histological analysis. Liver function, renal function indexes, and the number and distribution of blood cells were the same in the PBS group and MCFCNPs‐treated group, which indicated that the MCFCNPs were biocompatible (Figures S28 and S29, Supporting Information). Hematoxylin and eosin (H&E) staining did not reveal significant physiological or pathological tissue damage or inflammatory lesions in the organs, indicating the low cytotoxicity of the MCFCNPs (Figure S30, Supporting Information). Overall, we confirmed that the MCFCNPs have an excellent biosafety profile.

## 3 Conclusion

In summary, two new AIE‐active PSs that exhibited AIG‐ROS property were prepared by a simple process. Introduction of a thiophene bridge into the structure of PI boosted ROS production efficiency via the heavy atom effect and extended the emission wavelength to a valuable region for biological applications. Fluorescence imaging in vitro revealed a desirable and specific LD‐targeting ability of the PSs to lighten the cells. Additionally, the mechanism underlying PDT in the LDs was found to be ferroptosis caused by singlet oxygen species oxidizing PUFAs to form toxic LPOs. After coating PTI aggregates with MCF‐7 cell membranes to form MCFCNPs, the MCFCNPs exhibited a better tumor‐targeting ability and a higher ROS production efficiency, which resulted in an improved therapeutic efficacy in the MCF‐7 tumor‐bearing nude mice.

This work is the first to provide a comprehensive solution for increasing PDT efficiency of AIEgens. We have also discussed the therapeutic mechanism of the PSs and how to improve targeting efficiency. Thus, the findings of this study would be beneficial for developing biomimetic NPs with AIG‐ROS activity and excellent functions for precision cancer treatment.

## Conflict of Interest

The authors declare no conflict of interest.

## Supporting information

Supporting Information

## Data Availability

Research data are not shared.
