# Supplementary material for: Trojan Horse‐Like Nano‐AIE Aggregates Based on Homologous Targeting Strategy and Their Photodynamic Therapy in Anticancer Application
Source: Adv Sci (Weinh). 2021 Oct 20;8(23):2102561. doi: 10.1002/advs.202102561 (PMC8655165; doi:10.1002/advs.202102561)
Supplement: Supplementary file 1 — Supporting Information [file ADVS-8-2102561-s001.pdf]

WILEY-VCH

© 2021 Wiley-VCH GmbH

ADVANCED SCIENCE
Open Access

Supporting Information

for *Adv. Sci.*, DOI: 10.1002/advs.202102561

Trojan Horse-like Nano AIE Aggregates Based on Homologous Targeting Strategy and Their Photodynamic Therapy in Anticancer Application

Yin Li, Rongyuan Zhang, Qing Wan, Rong Hu, Yao Ma, Zhiming Wang, Jianquan Hou*,Weijie Zhang,* Ben Zhong Tang,**

Supporting Information

Trojan Horse-like Nano AIE Aggregates Based on Homologous Targeting Strategy and Their Photodynamic Therapy in Anticancer Application

Yin Li, Rongyuan Zhang, Qing Wan, Rong Hu, Yao Ma, Zhiming Wang Weijie Zhang,* Jianquan Hou*, Ben Zhong Tang**

Materials and Methods

All the solvents and reagents used in this work were of analytical grade. Phenothiazine, iodobenzene and 1, 3-indenedione were purchased from Energy Chemical Co., Ltd. 5-Formyl-2-thiopheneboronic acid was obtained from Soochiral Chemical Science & Technology Co., Ltd.. The biological chemical reagents Vitamin E was obtained from Sigma-Aldrich. Oleic acid (OA), phosphatidylcholine (PC), 9, 10-anthracenediyl-bis (methylene)-dimalonic acid (ABDA) and 2', 7'-dichlorodihydrofluorescein diacetate (H2DCF-DA) were offered from aladdin Co.. Cell Counting Kit-8(CCK-8) and liperfluo were obtained from Dongren Chemical Technology (Shanghai) Co., Ltd.. GPx4 antibody, β-actin antibody, Anti-EpCAM, Anti-Galectin-3 and Na^+ K^+ATPase antibody were purchased from Abcam Co., Ltd. Total GSH and GPx4 detection kits were purchased from Biyuntian Biotechnology Co., Ltd.

^{1}H and ^{13}C NMR spectra were measured on a Bruker AV 500 spectrometer. UV-Vis absorption spectrum was measured on a Shimadzu UV-2600 spectrophotometer. PL spectra were recorded on a Horiba Fluoromax-4 spectrofluorometer. Fluorescence quantum yields were measured using a Hamamatsu absolute PL quantum yield spectrometer C11347 Quantaurus_QY. Mass spectrometric (MS) data were carried out using LTQ Orbit rap XL instruments. Confocal laser scanning microscope (CLSM) images were performed on Olympus FV1000-IX81 confocal laser scanning microscope. Small animals' fluorescence imaging was carried out by NightOWL II LB983 living imaging system. The electronic structures were calculated by (time-dependent) density functional theory ((TD) DFT) at the level of M06-2X/6-31G (d, p). All the calculations were performed using Gaussian09 package.

Synthesis of 10-phenyl-10H-phenothiazine (1)

A mixture of phenothiazine (6 g, 30 mmol), copper powder (1.9 g, 30 mmol), potassium carbonate (8.3g, 60 mmol) and 18- crown ether -6 (3 g, 60 mmol) in N-dimethylformamide (60 ml) under nitrogen environment heated to 180 °C for 8 hours. The dichloromethane was extracted and dried with anhydrous magnesium sulfate , the crude product was purified on a silica-gel column. A colorless crystal (4.2g) was obtained in 60% yield. ^{1}H NMR (500 MHz, $CDCl_3$), 7.60 (t, 2H), 7.46 (t, 1H), 7.38 (d, 2H), 7.01 (d, 2H), 6.82 (m, 4H), 6.20 (d, 2H).

Synthesis of10-phenyl-10H-phenothiazine-3-carbaldehyde (2)

Add ultra dry N, N-dimethylformamide (2.8 mL, 7.2mmol) and 1,2-dichloroethane (5 mL) to 100 ml dry round bottom flask at 0 ℃, cool for ten minutes, and inject phosphorus oxychloride (2.1 mL, 4.5mmol) into the mixture drop by drop. Then 10-phenyl-10h-phenothiazine (1 g, 3.6 mmol) dissolved in 5 mL of 1,2-dichloroethane solution was slowly added in 30 minutes, heated to 90 °C for 12 hours, stopped the reaction and cooled to room temperature, then the mixture was poured into 100 mL of ice water, neutralized with saturated $NaHCO_3$ solution to pH = 7, The dichloromethane was extracted and dried with anhydrous magnesium sulfate , the crude product was purified on a silica-gel column. 0.6 g of golden yellow solid was obtained and the yield was 55%.^{1}H NMR (500 MHz, CD_2Cl_2), 9.62 (s, 1H), 7.58 (t, 2H), 7.49 (t, 1H), 7.40 (d, 1H), 7.32 (m, 2H), 7.21 (m, 1H), 6.9 (m, 1H), 6.76 (m, 1H), 6.05 (d, 1H), 6.03 (d, 1H).

Synthesis of 2-((10-phenyl-10H-phenothiazin-3-yl)methylene)-1H-indene-1,3(2H)-dione (PI) A mixture of 10-phenyl-10h-phenothiazine-3-aldehyde (0.46g, 1.5mmol) and 1,3-indenedione (0.33g, 2.25mmol), 0.5 mL of tetramethylammonium hydroxide in 40 mL of anhydrous ethanol under u nitrogen environment heated to 90°C overnight for reaction. The crude product is separated and purified by column analysis to obtain the purplish red compound (0.3g), the yield is 46% . ^{1}H NMR (500 MHz, $CDCl_3$), 8.38 (s, 1H), 7.98 (m, 2H), 7.94 (d, 1H), 7.77 (d, 2H), 7.75 (m, 2H), 7.67 (t, 1H), 7.38 (d, 2H), 7.00 (d, 1H), 6.83 (d, 2H), 6.11 (m, 2H). ^{13}C NMR (126 MHz, $CDCl_3$) δ 191.04, 189.62, 159.58, 145.46, 142.55, 140.14, 135.55, 135.12, 134.87, 132.34, 131.34, 130.83, 129.27, 127.13, 126.83, 123.89, 123.12, 123.09, 116.66, 115.25.HRMS (m/z):431.1023.

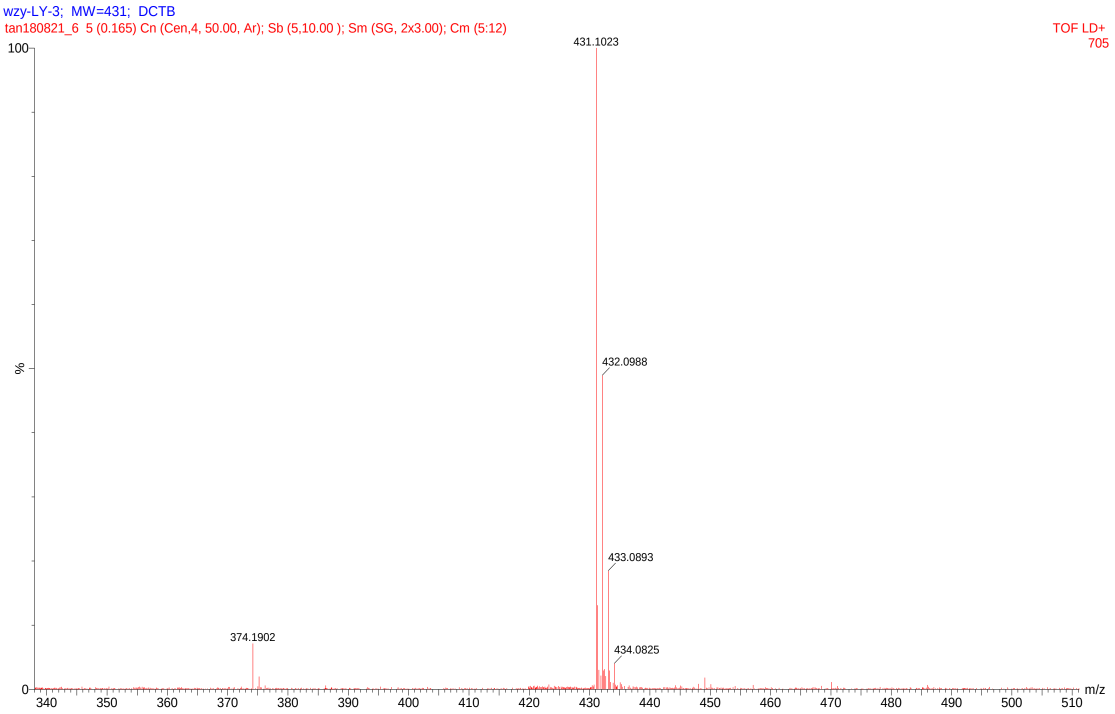

Synthesis of 3-bromo-10-phenyl-10H-phenothiazine (3)

Compound (1) (3 g, 10.91 mmol) was dissolved in 80 mL of DMF (20 mL) under ice cooling, and then the N-bromosuccinimide (NBS)(1.93 g, 10.91mmol) dissolved with DMF (10 mL) was added to the reaction bottle drop by drop. Return to room temperature and reaction for 2 h. After the reaction, the mixture was extracted by DCM. The solvent was removed by vacuum distillation to give light cyan crystal (3 g), the yield is 78%.^{1}H NMR (500 MHz, $CDCl_3$) 7.61 (1 H, t, J 7.8), 7.49 (1 H, t, J 6.9), 7.37 (1 H, dd, J 8.3, 1.1), 7.11 (1 H, d, J 2.3), 6.99 (1 H, dd, J 7.3, 1.8), 6.90 (1 H, dd, J 8.8, 2.3), 6.86 – 6.76 (1 H, m), 6.17 (1 H, dd, J 8.0, 1.4), 6.02 (1 H, d, J 8.8).

Synthesis of 5-(10-phenyl-10H-phenothiazin-3-yl)thiophene-2-carbaldehyde (4)

A mixture of 3-bromo-10-phenyl-10H-phenothiazine (1g, 2.84mmol), (5-formylthiophen-2-yl)boronic acid (513.2mg, 3.4mol), tetrakis(triphenylphosphine)palladium(0) ($Pd(PPh_3)_4$) (656.3mg, 0.56mmol) and potassium carbonate (1.2g, 8.68mmol)were added into a 100 mL two-neck flask under nitrogen atmosphere. Subsequently, a mixed solvent system of THF and water(v/v=5:1) 24mL was injected into the bottle, heatd to 90°C for 12 hours. After cooling to room temperature, extract with DCM, and the orange crystal (150mg) was obtained by a silica-gel column, the yield is 14%.^{1}H NMR (500 MHz, $CDCl_3$) δ 9.83 (s, 1H), 7.69 – 7.61 (m, 3H), 7.52 (t, J = 7.4 Hz, 1H), 7.37 (dd, J = 12.0, 7.3 Hz, 2H), 7.29 (s, 2H), 7.22 (d, J = 3.9 Hz, 1H), 7.11 (d, J = 2.3 Hz, 1H), 7.01 (d, J = 7.9 Hz, 1H), 6.89 (dd, J = 26.7, 17.9 Hz, 1H), 6.05 (dd, J = 72.8, 8.7 Hz, 2H). ^{13}C NMR (126 MHz, $CDCl_3$) δ 182.77, 141.71, 137.76, 134.49, 131.35, 131.21, 129.83, 129.16, 128.91, 127.18, 125.52, 125.27, 123.21, 123.19, 117.27, 116.17, 116.09, 115.96.

Synthesis of 2-((5-(10-phenyl-10H-phenothiazin-3-yl)thiophen-2-yl)methylene)-1H-indene-1,3(2H)-dione (PTI)

Add compound 4 (170mg, 0.45mmol), 1.3-indenedione (72.5mg, 0.5mmol) and p-Toluenesulfonic acid (5.17mg, 0.03mmol) into two neck bottles in nitrogen atmosphere, then inject 20mL toluene, heat to reflux for 24 hours. After cooling to room temperature. After solvent evaporation, the purple solid (50 mg) was obtained by a silica-gel column, the yield is 22%.^{1}H NMR (500 MHz, $CDCl_3$) δ 7.93 (s, 2H), 7.89 (s, 2H), 7.76 – 7.73 (m, 2H), 7.64 (dd, J = 10.9, 4.6 Hz, 2H), 7.53 (dd, J = 10.7, 4.3 Hz, 1H), 7.41 – 7.36 (m, 3H), 7.27 (s, 1H), 7.21 – 7.18 (m, 1H), 7.00 (dd, J = 10.3, 6.5 Hz, 1H), 6.90 – 6.78 (m, 2H), 6.13 (d, J = 7.4 Hz, 2H). ^{13}C NMR (126 MHz, $CDCl_3$) δ 190.55, 189.86, 143.89, 142.13, 140.59, 136.43 – 134.91, 134.97 – 134.91, 134.85, 131.30, 129.78, 129.17, 128.94, 127.08, 125.49, 123.91, 123.62, 122.96, 117.31, 116.27, 116.04, 115.93. HRMS (m/z):513.0851.

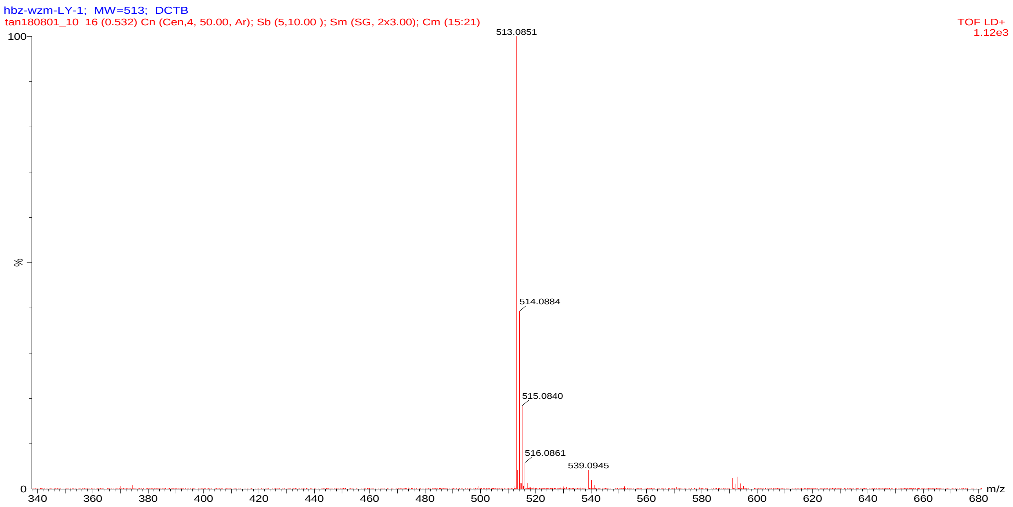

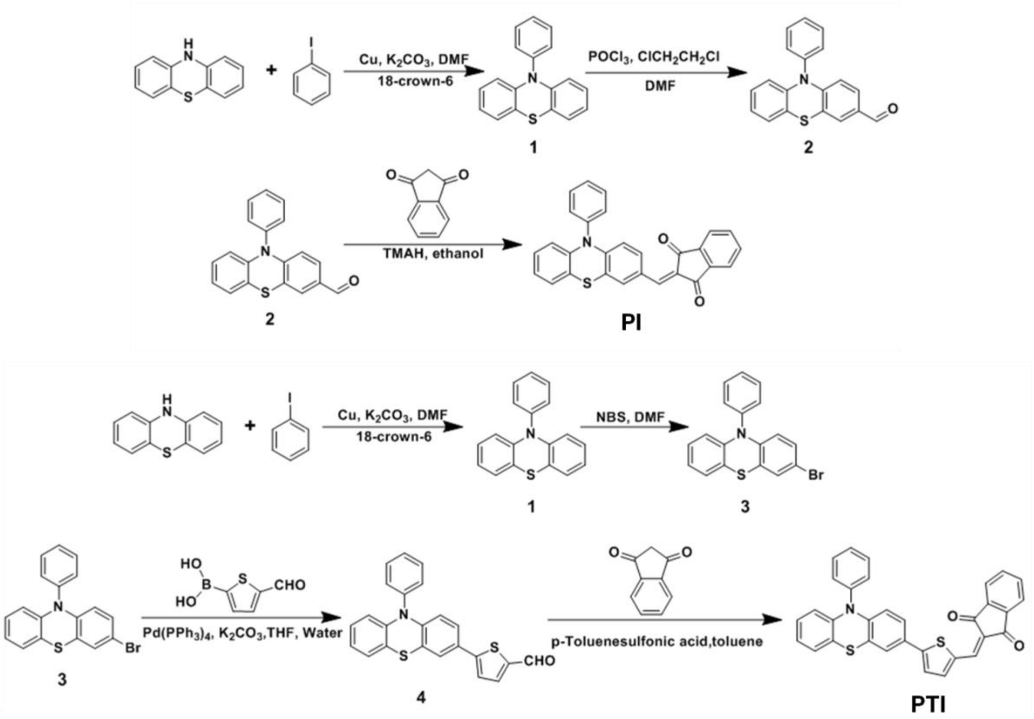

Figure S1 Synthetic route of PI and PTI.

Extracellular lipid peroxidation model and mass spectrometry analysis

First, prepare oleic acid (OA) and phosphatidylcholine (PC) into a 200 μM aqueous solution, and PI and PTI molecules into a 100 μM aqueous solution. Then, we mix OA solution (250 µL) with PI (250 µL) and PTI (250 µL) solutions respectively. Similarly, mix PC and photosensitizers in the same proportion. The above four groups of samples are experimental groups, and pure OA and PC solution are used as control groups. The sample was continuously irradiated with white light (50 mW/cm^2) for 30 min, then the mass spectrum data of each sample was collected, and the molecular weight changes of OA and PC were analyzed.

MCF-7 cell membrane extraction

In order to prepare sufficient MCF-7 cell membranes, we use 100 mm^2 culture dishes to culture cells at 37 ^{o}C and 5% CO_2, and then separated the cells with a cell scraper. Next, centrifuge the separated cells for 10 min at 500g at 4 ^{o}C, remove the supernatant, add pre-cooled homogenization buffer at 4 ^{o}C to resuspend the cells; Then use the hand-held dounce homogenizer to crush the cells 20~30 times on ice repeatedly. Through a centrifugation treated at 500g for 10 min at 4 ^{o}C, resuspending the collected cells into pre-cooled homogenization buffer and break again, repeating the above operation until no complete cells can be found under the microscope. Add the collected supernatant to a concentration gradient glucose physiological saline solution (30 %, 40 %, 55 %) and ultracentrifuge at 28000 g at 4°C for 60 min. Finally, the cell membrane is located at the intersection of 30% and 40% interface, collect the supernatant, and freeze-dry it overnight in a lyophilizer.[1]

The manufacturing procedures of MCFCNPs

The manufacturing of MCFCNPs consists of the following procedures: poly(lactic-co-glycolic acid)(PLGA) (0.5mg) and PTI (2.0mg) were dissolved in acetonitrile (1.5mL) respectively, and then mixed uniformly after 5 minutes of sonication in a water bath, and 2 minutes of sonication in a water bath. The MCF-7 cell membrane (0.5mg) was dissolved in ultrapure water (2.0mL), and ultrasonically vibrated for 2 minutes with a probe type ultrasonic cell disruptor (20% power, working 3s, interval 1s), and then water bath ultrasonic (power 100%, 80HZ) for 5 minutes to get MCF-7 cell membrane solution. Dispersed the mixture of PTI and PLGA in ultrapure water, and ultrasonic (power 100%, 80 HZ) in a water bath for 10 min. After the acetonitrile evaporated, freeze-dry it in a lyophilizer. Through a centrifugation treated at 4200g for 10 min at 4 ^{o}C, removed the precipitation and saved the supernatant, then dark red PLGA core nanoparticles were obtained after filtration. Finally, MCF-7 cell membrane solution was added to PLGA nuclear solution drop by drop under stirring. After 5 minutes of ultrasonic bath, almost all PLGA cores had been coated on the cell membrane, the concentration of MCFCNPs is 1mg/mL after concentration.

Animals and tumor models

All animal experiment protocols were approved by the Animal Ethics Committee of South China University of Technology. Female SPF BALB/c Nude mice aged 3 to 4 weeks were purchased from Guangdong Medical Experimental Animal Center and raised to six weeks in the Animal Experimental

Center of South China Agricultural University. Balb/c nude mice are used to establish mouse models of breast cancer. In short, 1×10^7 MCF-7 breast cancer cells were injected subcutaneously into selected locations to establish a breast cancer tumor model in Balb/c nude mice. The tumor is allowed to grow to a volume of about 50 mm^3 before being used for in vivo imaging and photodynamic therapy.

***In vivo* PDT assessment**

In order to test the PDT efficiency of MCFCNPs *in vivo*, we studied their anti-tumor properties *in vivo* by intratumor administration. Divide all thirty mice into six groups, each with five mice: group I (negative control group), grow naturally; group II (PBS tail intravenous injection and intratumor injection with light group, ie PBS+L); group III (MCFCNPs intratumor injection and tail intravenous injection without light group, ie MCFCNPs-L); group IV(MCFCNPs intratumor injection with light group, ie MCFCNPs Tumoral injection + L); group V (MCFCNPs tail intravenous injection with illumination group, ie MCFCNPs Intravenous injection + L); group VI (MCFCNPs combined intratumoral injection and tail intravenous injection with illumination group, ie MCFCNPs Combination injection + L). The concentration of MCFCNPs is 1 mg/mL, and the dose for tail intravenous injection is 200 µL/mouse, and the dose for intratumoral injection is 30 µL/mouse. When MCF-7 heterotopic transplantation tumor is treated with photodynamic therapy, the light source adopts a deuterium lamp, the wavelength range of white light is 400~1000 nm, and the energy density is 200 mW/cm^2; Group II, Group IV, Group V and Group VI received three PDT treatments. During the treatment period, the tumor volume of all mice was measured with vernier calipers every two days. Then, the maximum longitudinal diameter (length) and the maximum lateral diameter (width) are used to calculate the tumor volume. Tumor volume $V = length\times width^2/2$. 14 days after treatment, the tumors of all groups were collected and weighed. For histological analysis, hematoxylin-eosin (H&E) staining was performed on tumor sections.

Table S1 The photophysical properties of two compounds

^{a}Maximum absorption wavelength, concentration: 10×10^{-6} M ; ^{b} Maximum emission wavelength, soln: THF solution; ^{c}absolute fluorescence quantum efficiency ;

Compounds	$\lambda_{abs}{}^{a}$ (nm)	$\lambda_{em}{}^{b}$ (nm)		Φ^c (%)	
	soln	soln	film	soln	film
PI	509	667	746	1.7	4.8
PTI	500	700	813	0.6	1.3

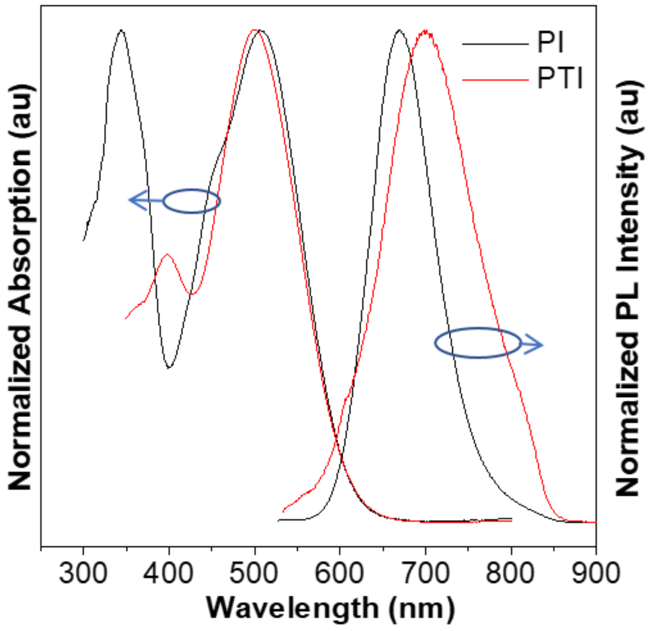

Figure S2 Absorption and PL spectra of PI and PTI in THF. (Concentration: 10×10^{-6} M)

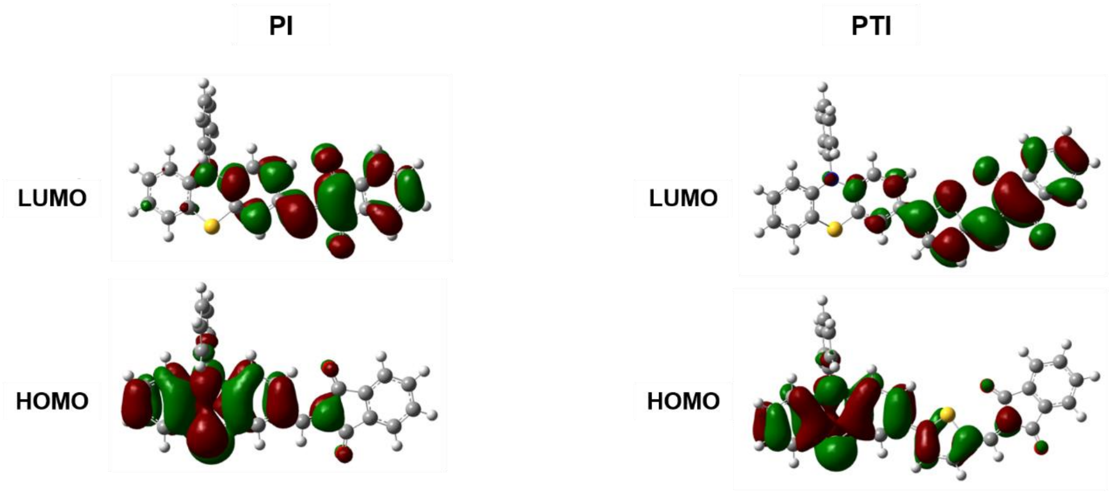

Figure S3 DFT simulation of HOMO and LUMO energy levels of PI (C) and PTI (D) based on the M06-2X/6-31G (d, p) level.

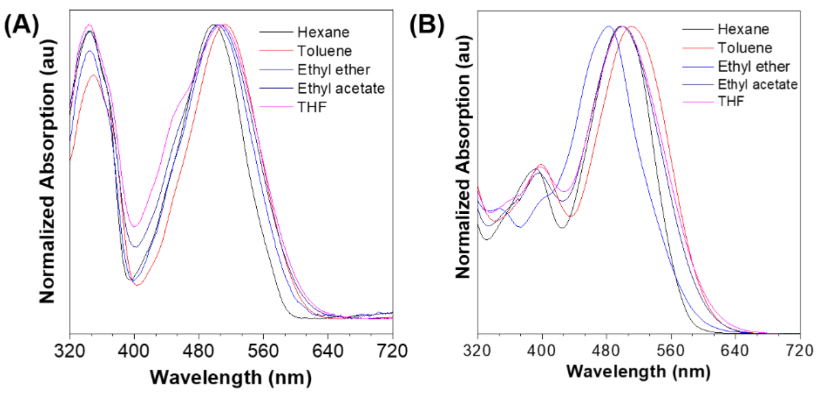

Figure S4 UV-Vis spectra (A) PI and (B) PTI in solvents with different polarity (Concentration: 10×10^{-6} M) .

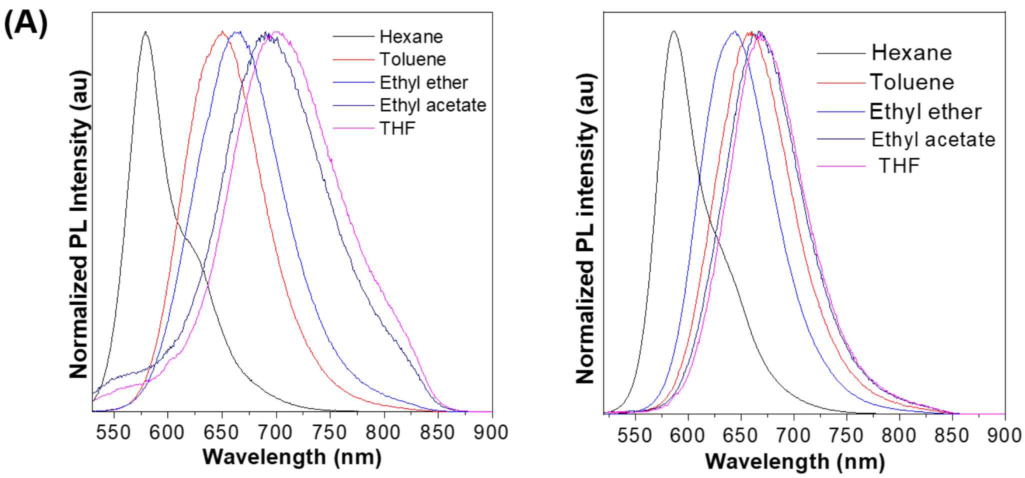

Figure S5 PL spectra of PI (A) and PTI (B) in solvents with different polarity (Concentration: 10×10^{-6} M) .

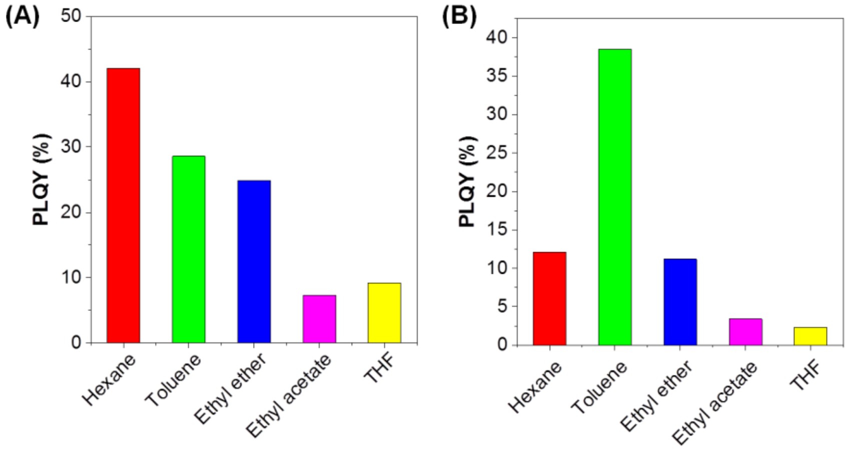

Figure S6 The photoluminescent quantum yields (PLQYs) of PTI in solvents with different polarity (Concentration: 10×10^{-6} M) .

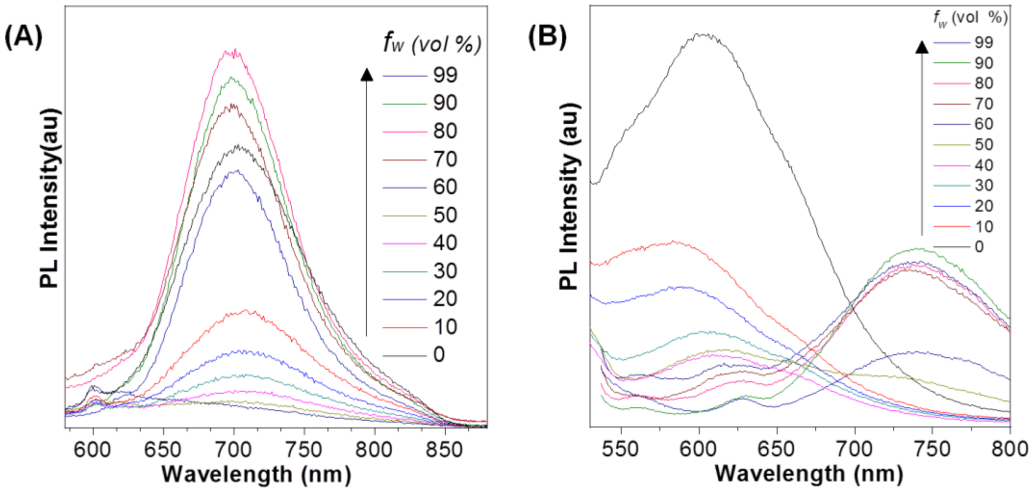

Figure S7 PL spectra of (A) PI and (B) PTI in DMF/water mixtures with different water fractions (Concentration: 10×10^{-6} M).

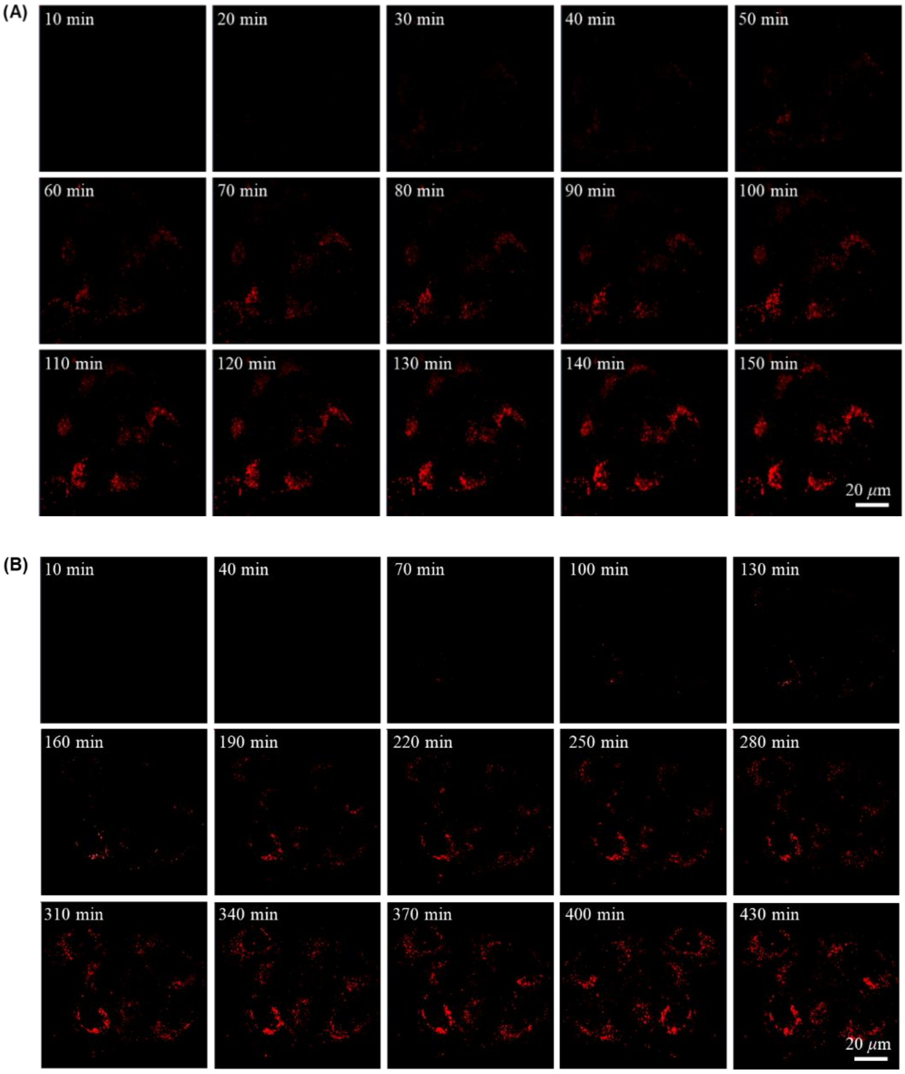

Figure S8 CLSM images of MCF-7 cells stained with PI (A) and PTI (B) at different time; Ex = 543 nm, Em = 630-720 nm, Laser power 5%. Scale bar = 20 μm (Concentrations: 15×10^{-6} M (AIEgens)).

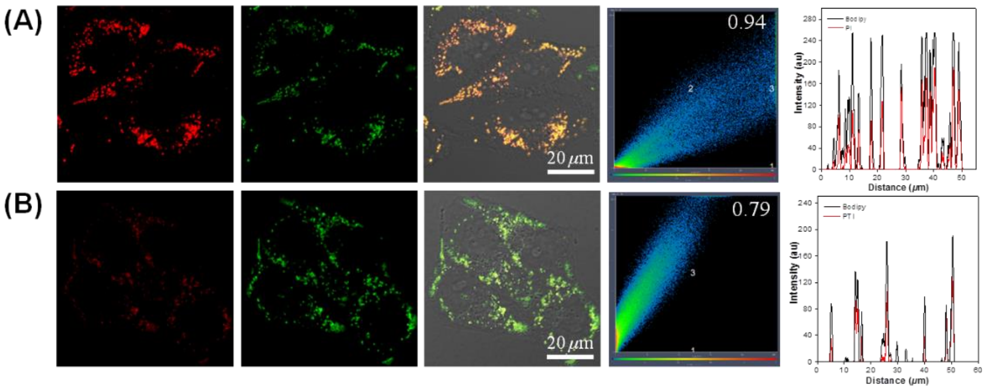

Figure S9 Colocalization imaging of MCF-7 cells stain with BODIPY and AIEgens (A)PI and (B)PTI; Ex = 543 nm, Em = 630-720 nm, Laser power 5%; Bodipy: Ex = 488 nm, Em = 500-550 nm, Laser power 2%. Scale bar = 20 μm (Concentrations: 10×10^{-6} M (AIEgens) and 2×10^{-6} M (BODIPY)).

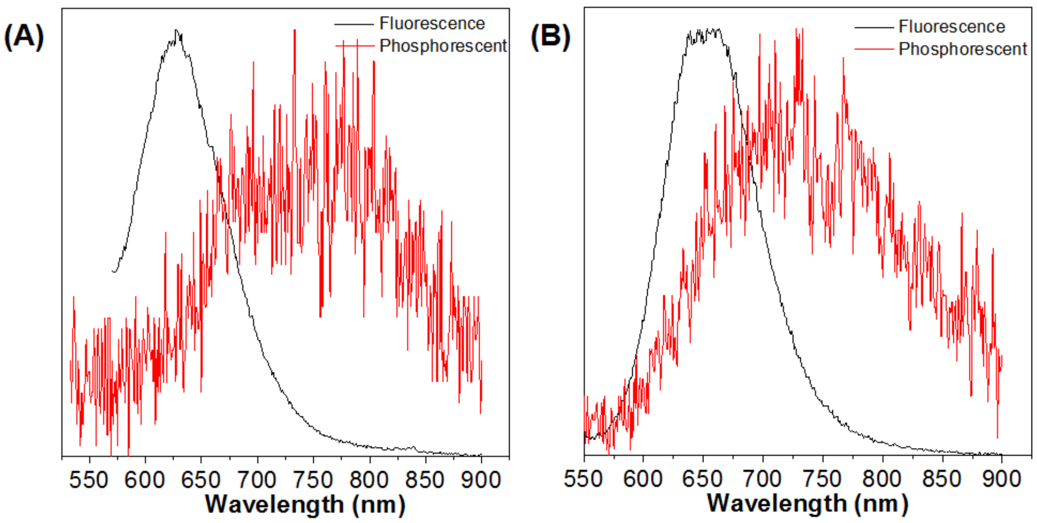

Figure S10 The low-temperature fluorescence and phosphorescence spectra of PI (A) and PTI (B) in DMF (concentration: 10×10^{-6} M).

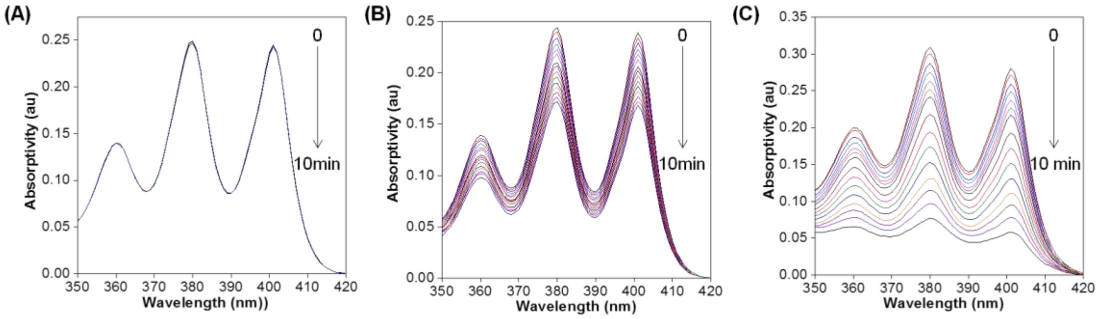

Figure S11 Absorbance intensity of ABDA (A) black, after photodecomposition by ROS upon white light irradiation of PBS (B), PI (C), Concentrations: 10×10^{-6} M (AIEgens) and 40×10^{-6} M (ABDA).

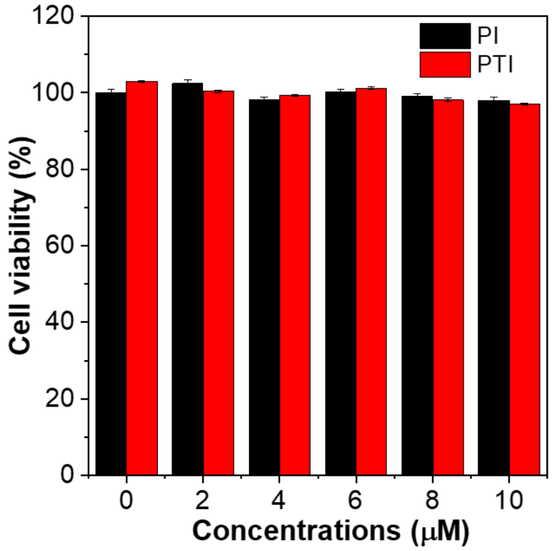

Figure S12 Cell viability of MCF-7 cells incubated with different concentrations of PI and PTI without white light irradiation (50 mW/cm^2).

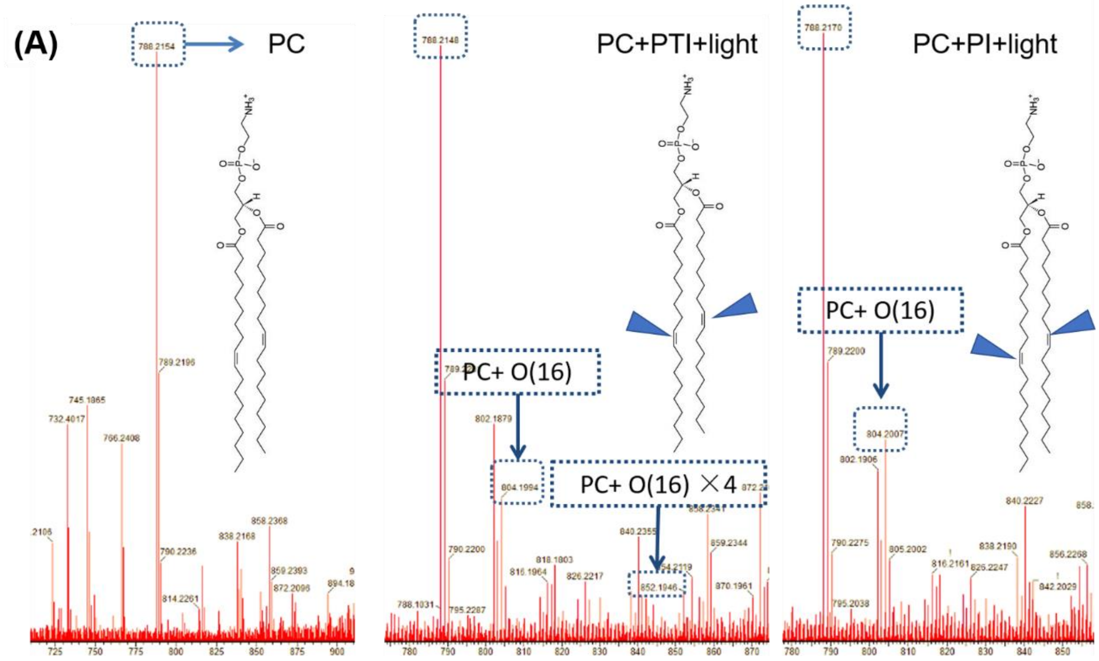

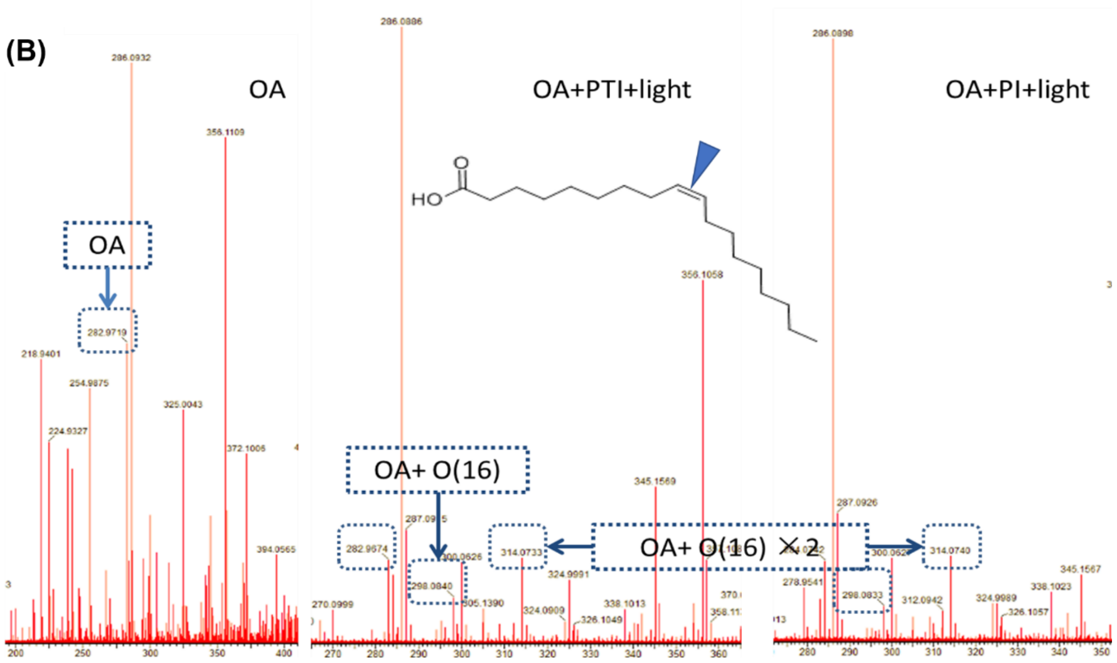

Figure S13 The mass spectrometry analysis of (A) PC and (B) OA oxidized by PI and PTI, (white light irradiation: 50 mW/cm^2).

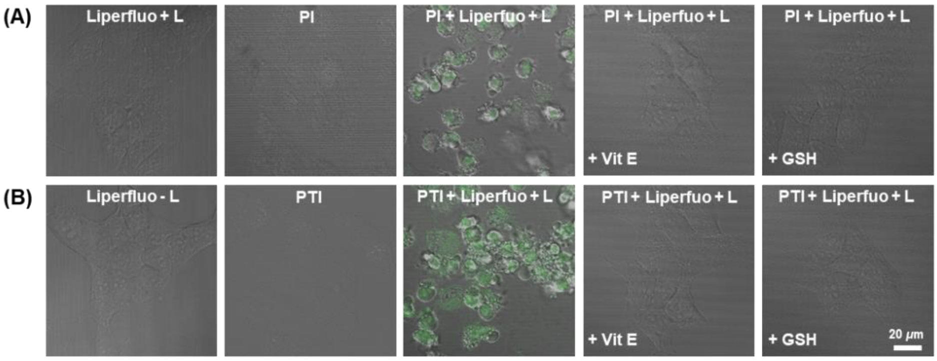

Figure S14 Confocal images of LPOs in MCF-7 cells detected by Liperfluo (A) PI and (B) PTI; scale bar = 20 μm.

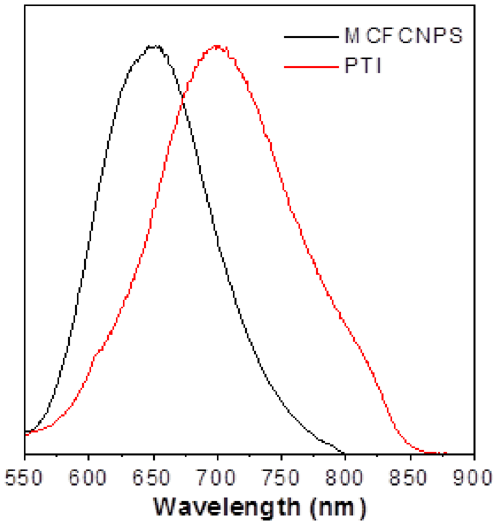

Figure S15 The PL spectra of PTI and MCFCNPs.

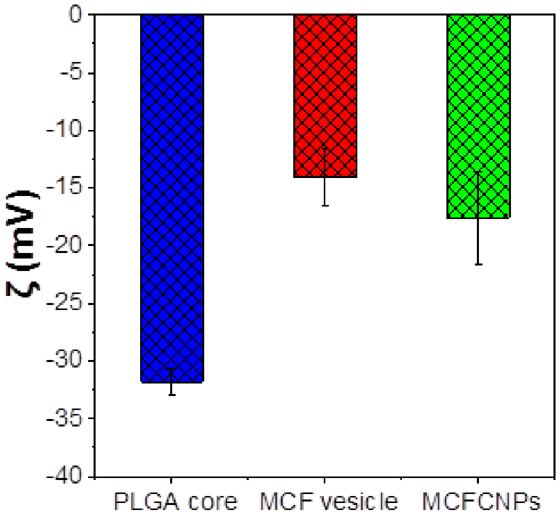

Figure S16 Zeta potential of PLGA core, MCF-7 cell membrane vesicles and MCFCNPs.

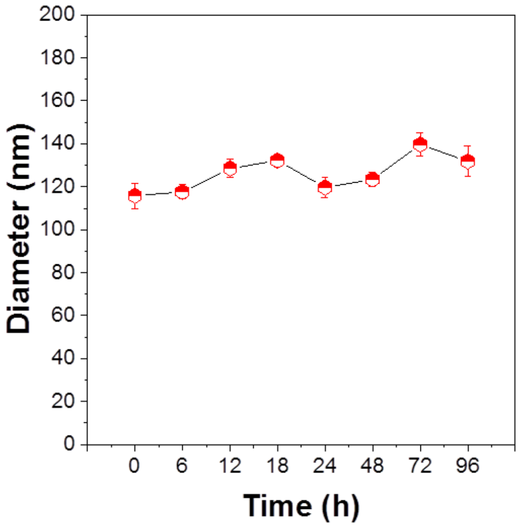

Figure S17 Time dependent curve of particle size of MCFCNPs in PBS.

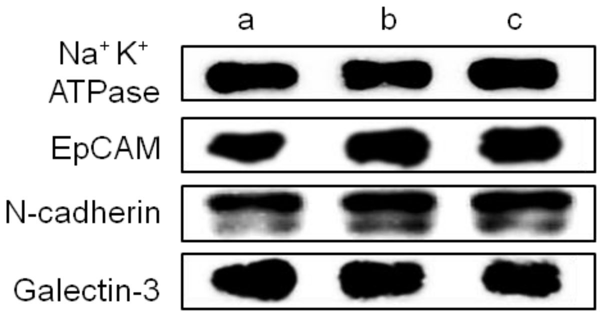

Figure S18 Western blotting analysis of membrane-specific protein markers. Samples are run at equal protein concentrations and immune-stained against membrane markers including EpCAM, N-cadherin, Galectin-3 and and Na+/K+-ATPase. a: MCF-7 cell lysate; b: MCF-7 cell membrane vesicle; c:MCFCNPs.

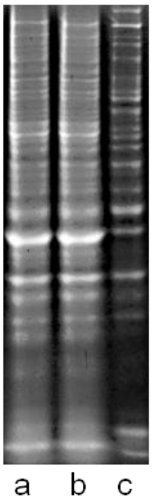

Figure S19 Protein analysis. Samples are stained with Coomassie Blue. a: MCF-7 cell lysate; b: MCF-7 cell membrane vesicle; c: MCFCNPs.

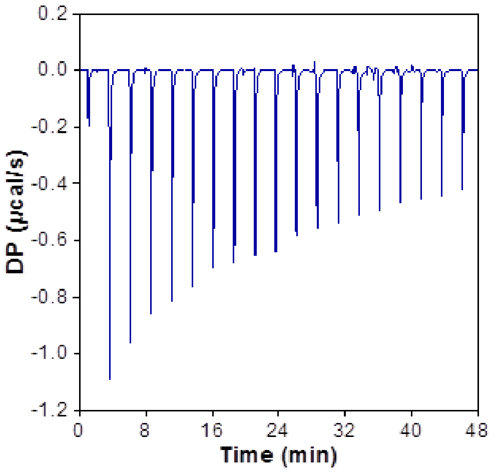

Figure S20 Isothermal titration calorimetric curve of MCF-7 cell membrane vesicles binding to PLGA cores.

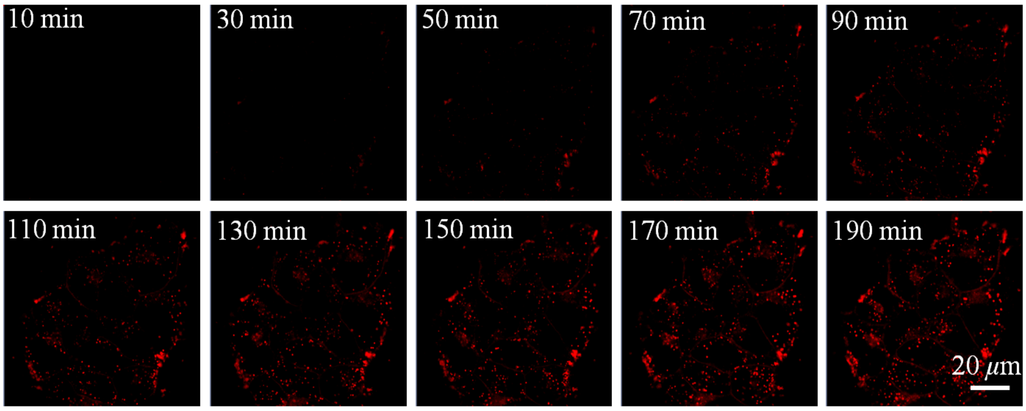

Figure S21 Confocal images of MCF-7 cells treat with MCFCNPs for different time points. Ex = 543 nm, Em = 630-720 nm, Laser power 10%. Scale bar = 20 μm, Concentrations: 10×10^{-6} M.

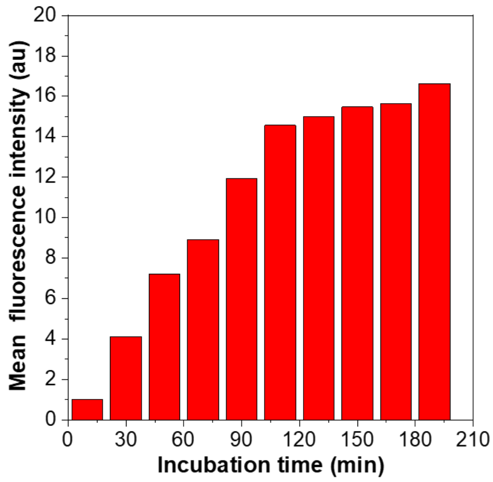

Figure S22 MFI of MCF-7 cells coincubation with MCFCNPs for different time points. Ex = 543 nm, Em = 630-720 nm, Laser power 10%. Scale bar = 20 μm, Concentrations: 10×10^{-6} M.

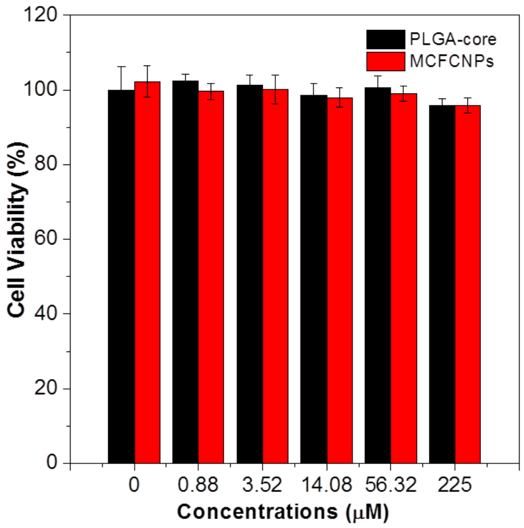

Figure S23 Viability of cells of MCF-7 breast cancer cells without white light irradiation, which incubated with different concentrations of PLGA cores and MCFCNPs for 12h.

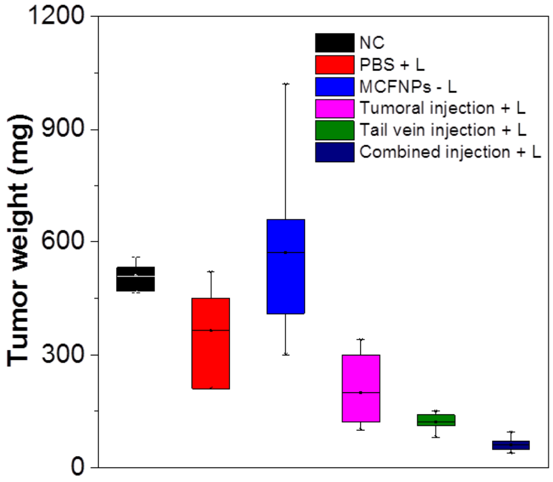

Figure S24 Quantitative tumor weight in each group.

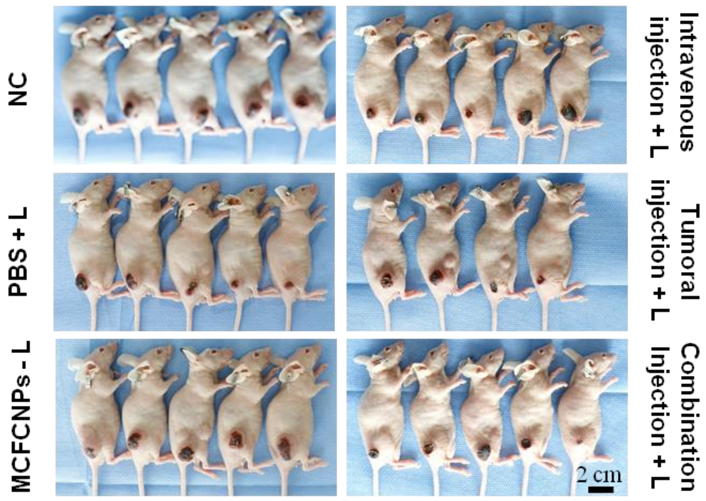

Figure S25 Representative photos of mice bearing MCF-7 tumors.

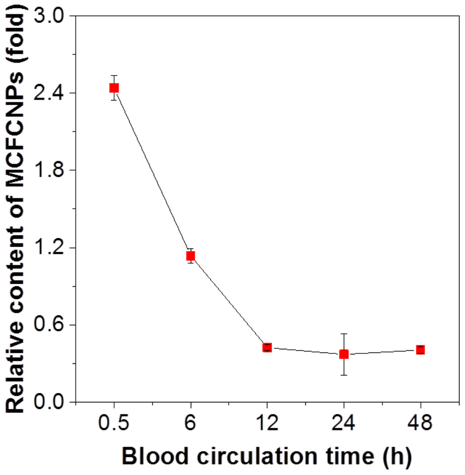

Figure S26 Time curve of blood circulation in the body of healthy Balb/c mice intravenously injected with MCFCNPs for 2 days.

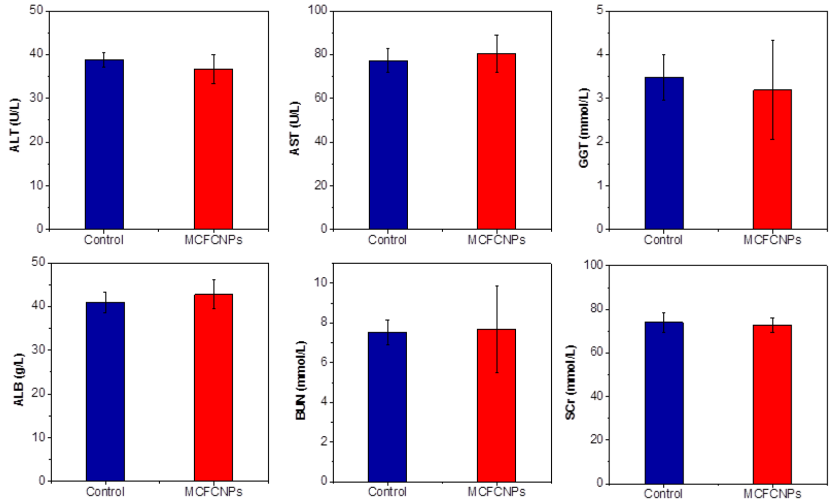

Figure S27 Blood biochemistry test of the healthy mice with and without tail intravenous injection of MCFCNPs.

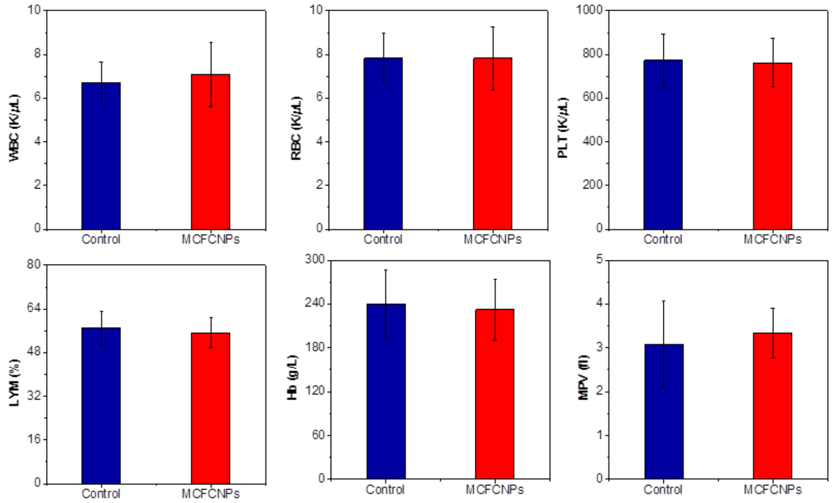

Figure S28 Blood routine assays of the healthy mice with and without tail intravenous injection of MCFCNPs.

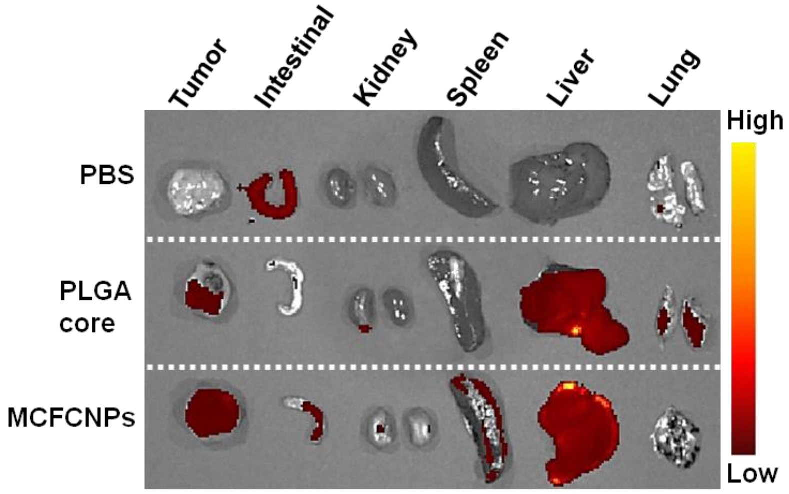

Figure S29 Fluorescence imaging of isolated tumors and organs (intestinal, kidney, spleen, liver, and lung) in different experimental groups.

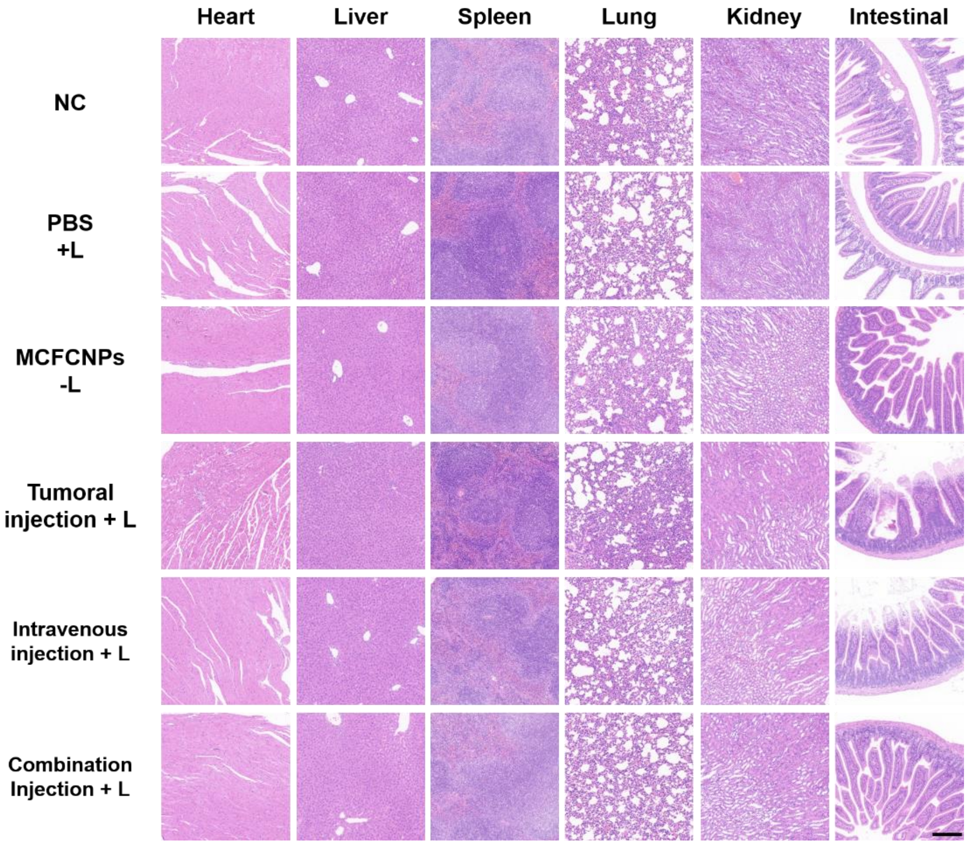

Figure S30 H&E staining of major organs of mice from different treatment groups after 14 days of treatment. Scale bar = 200 μm for all images.

^{1}H NMR and ^{13}C NMR spectrum

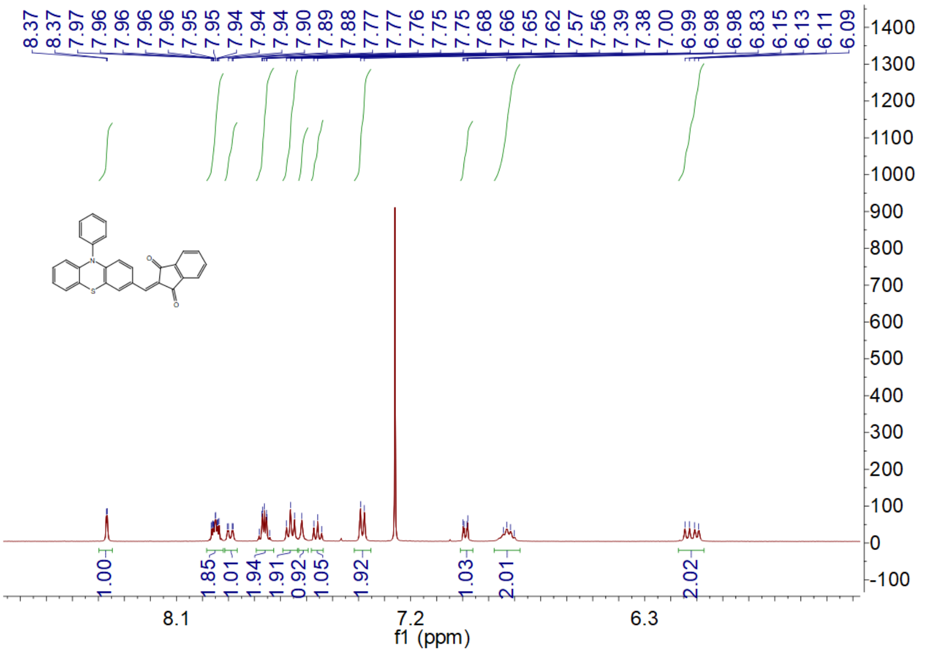

Figure S31 ^{1}H NMR spectrum of PI.

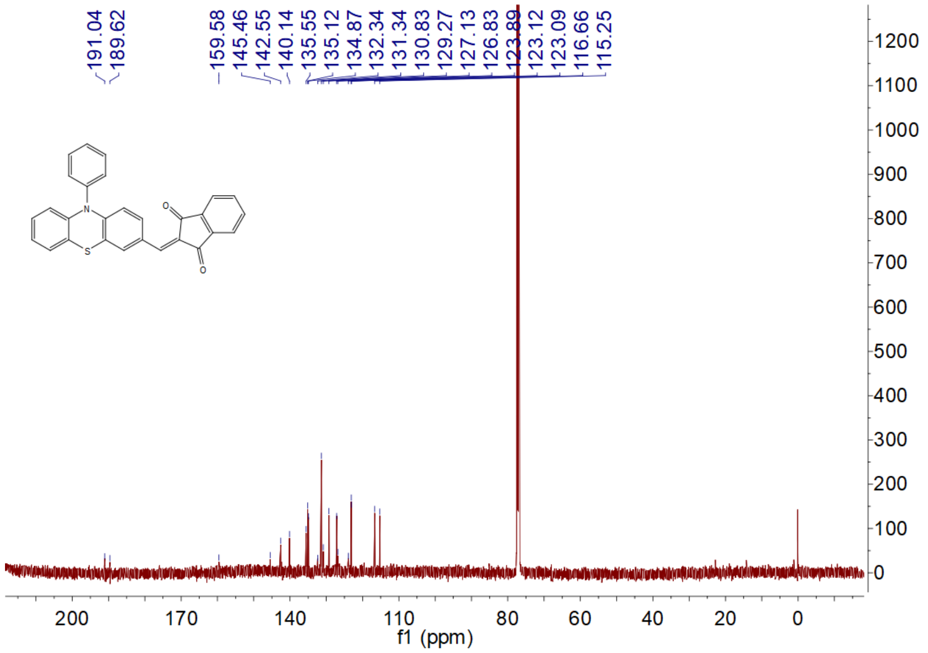

Figure S32 ^{13}C NMR spectrum of PI.

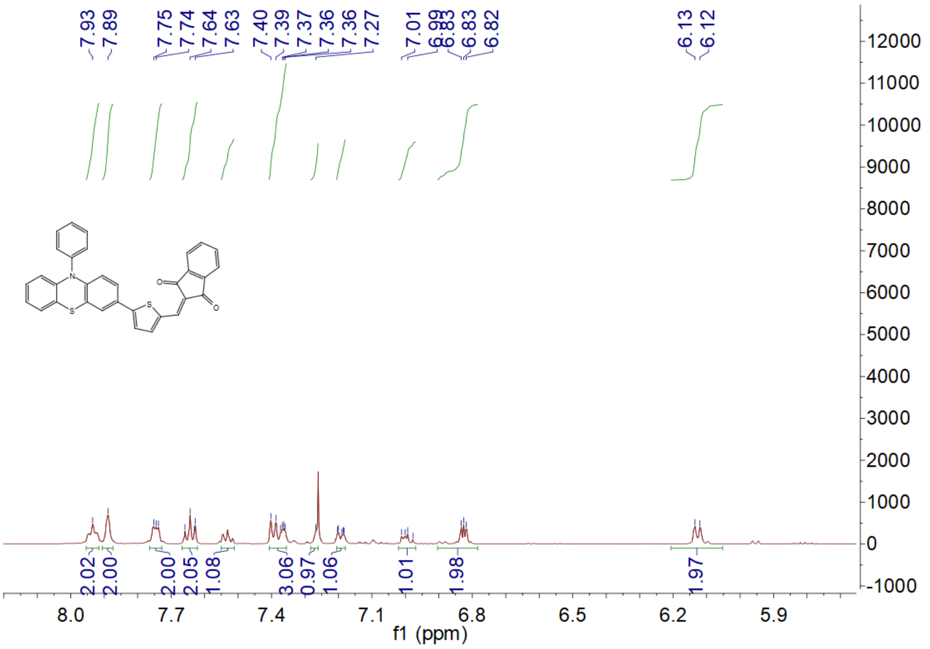

Figure S33 ^{1}H NMR spectrum of PTI.

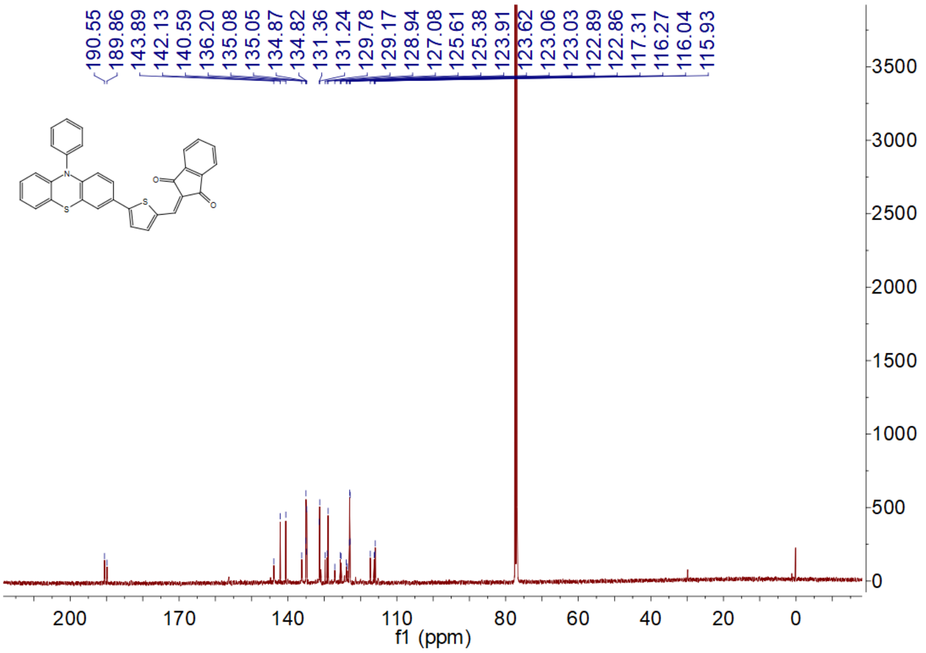

Figure S34 ^{13}C NMR spectrum of PTI.

REFERENCES

[1] Naito, S., Von Eschenbach, A. C., Giavazzi, R., & Fidler, I. J. *Cancer Res*. **1986**, *46*, 4109.
